# Teaching handwashing with soap for schoolchildren in a multi-ethnic population in northern rural Vietnam

**DOI:** 10.3402/gha.v6i0.20288

**Published:** 2013-04-24

**Authors:** Le Thi Thanh Xuan, Thilde Rheinländer, Luu Ngoc Hoat, Anders Dalsgaard, Flemming Konradsen

**Affiliations:** 1 Department of Environmental Health, Institute for Preventive Medicine and Public Health, Hanoi Medical University, Hanoi, Vietnam; 2 Department of International Health, Immunology and Microbiology, University of Copenhagen, Copenhagen, Denmark; 3 Department of Biostatistics and Medical Informatics, Institute for Preventive Medicine and Public Health, Hanoi Medical University, Hanoi, Vietnam; 4 Department of Veterinary Disease Biology, University of Copenhagen, Copenhagen, Denmark

**Keywords:** handwashing with soap, schoolchildren, multi-ethnic, Vietnam

## Abstract

**Background:**

In Vietnam, initiatives have been started aimed at increasing the practice of handwashing with soap (HWWS) among primary schoolchildren. However, compliance remains low.

**Objective:**

This study aims to investigate responses to a teacher-centred participatory HWWS intervention in a multi-ethnic population of primary schoolchildren in northern rural Vietnam.

**Design:**

This study was implemented in two phases: a formative research project over 5 months (July–November 2008) and an action research project with a school-based HWWS intervention study in two rural communes during 5 months (May, September–December 2010). Based upon knowledge from the formative research in 2008, schoolteachers from four selected schools in the study communes actively participated in designing and implementing a HWWS intervention. Qualitative data was collected during the intervention to evaluate the responses and reaction to the intervention of teachers, children and parents. This included semi-structured interviews with children (15), and their parents (15), focus group discussions (FGDs) with schoolchildren (32) and school staff (20) and observations during 15 HWWS involving children.

**Results:**

Observations and interview data from children demonstrated that children were visibly excited and pleased with HWWS sessions where teachers applied active teaching methods including rewards, games and HWWS demonstrations. All children, schoolteachers and parents also viewed the HWWS intervention as positive and feasible, irrespective of ethnicity, gender of schoolchildren and background of schoolteachers. However, some important barriers were indicated for sustaining and transferring the HWWS practice to the home setting including limited emphasis on hygiene in the standard curriculum of schools, low priority and lack of time given to practical teaching methods and lack of guidance and reminding HWWS on a regular basis at home, in particular by highland parents, who spend most of their time working away from home in the fields. Access to soap and water at the household level did not seem a barrier for the uptake of HWWS but continuous access to these might be a challenge at schools.

**Conclusions:**

This study demonstrated that it is feasible to engage teachers and implement active teaching methods for behaviour change of HWWS in a group of multi-ethnic primary schoolchildren without the need for major investments in water and hygiene infrastructures. However, in those areas there was limited transfer of practice from school promotion to home. Continuous access to soaps at schools needs to be invested.

Handwashing with soap (HWWS) is considered one of the most cost-effective means of preventing faecal–oral transmitted diseases and other infections, especially in developing countries (1–5). Good habitual HWWS behaviour brings benefits for all, particularly for schoolchildren who are more impacted by the diseases (6–10).

A recent systematic review reflects that most research on outcome measures and maintenance of HWWS behaviour in low- to middle-income countries has focused on the community level and only a few studies have focused on school settings (11). However, a study, conducted as a cluster-randomized controlled trial among 87 Chinese primary schools, found that teaching HWWS at school was feasible and could be done at low cost (12). Several intervention studies have illustrated ‘double advantages’ of school hygiene promotion; being effective in reaching a large number of schoolchildren, and at the same time using children as ‘hygiene messengers’ to their families and communities (13–20). However, HWWS promotion methods have varied across studies without any conclusive suggestions to best choice, e.g. it remains to be investigated to what extent school-based participatory action research can be used to effectively promote HWWS (4).

Vietnam has given high political priority to the achievement of free and compulsory primary education for all (21). In 2009, the net enrolment rate in primary education in Vietnam was 97% (22), providing a significant opportunity to influence the hygiene behaviour of children and future adults by targeting the school setting. However, the current curriculum, which includes the topic of hygiene, is usually focused on increasing knowledge through lecturing. Therefore, language barriers have been highlighted as a constraint in teaching hygiene aimed at students from ethnic minority groups (EMGs) (23). In addition, in these areas, schoolchildren are often living in poor hygiene conditions and are facing risks of infectious diseases making hygiene promotion of particular relevance. Innovative approaches in teaching hygiene have thus been requested to effectively promote behaviour change for EMG schoolchildren (24–28). In multi-ethnic populations, the priority of practical teaching methods has been low and an increased effort is therefore needed to innovate school-based hygiene interventions (28, 29).

Compliance of HWWS among schoolchildren in Vietnam remains low, as documented by a nationwide survey on environmental sanitation at schools in 2007 (30). The survey showed that only 11.5% of students washed their hands with soap after defaecation and the situation was worse in northern Vietnam. Recently, some HWWS initiatives, including some in school-based settings, have been implemented in Vietnam. A school HWWS initiative by the World Bank emphasized the need to use materials and strategies combining education and entertainment in its campaign focusing on students of the Kinh ethnic majority (31). Another initiative followed a public–private-partnership (PPP) model focusing on a consumer-oriented approach aimed at households (32). Since 2009, Vietnam has also implemented an annual national handwashing (HW) day aimed at improving HW practices in all groups and settings.

Despite this recognition of the need for school-based hygiene promotion and new teaching approaches to promote HWWS, the effectiveness of behaviour change and HWWS interventions in school settings of multi-ethnic populations is largely unknown (33, 34). A student-centred hygiene approach has been shown to be effective in other studies (35–37) but changing priorities of staff might improve the effectiveness of educational hygiene teaching methods and interventions (38). In multi-ethnic populations, participatory teaching curricular and teaching cultures needs to develop if school and school staff are expected to support changes in HWWS behaviour for primary schoolchildren (28). This study was therefore conducted to investigate responses to a teacher-centred participatory HWWS intervention in schools with ethnically diverse schoolchildren in northern rural Vietnam. The findings can add to the limited knowledge about how to involve schools in designing and implementing active school-based hygiene interventions, including how to initiate HWWS behaviour change among schoolchildren and their families.

## Methods

### Field settings

The research was carried out in two rural communes, the local authoritative unit, in a Northern Province of Vietnam with a total population of approximately 10,000 people. The two communes were selected because they were pilot sites for hygiene promotion activities in the second phase (2006–2010) of the National Target Program for Rural Water Supply and Sanitation (NTP-RWSS II).

The primary school system in the two communes includes one main primary school (trường chính) located in the communal centre and 14 smaller primary branch schools (điểm trường) located in the surrounding villages. All schools in the selected communes are public schools and teachers are mainly from the majority ethnic group of Vietnam (the Kinh) and live outside the rural communes in urban settings, except for five head teachers (2 Tày, 2 Dáy and 1 from the Xa Phó ethnic group). In each main school, a student advisor (cô tổng phụ trách) is responsible for coordinating all collective activities for schoolchildren, including extra-curricular activities. In each branch school, a team leader is responsible for these same activities. Four primary schools were purposely selected for the intervention, including three schools located in the lowland (valleys with irrigated fields and within 2 kilometres of the communal centre) and one school located in the highland (hilly landscape located up to 15 kilometres away from the communal centre). Due to their location in villages of different ethnic groups, students in each of the four schools were mainly of the same ethnic minority (Tày, Dáy and Xa Phó; Table 1).

**Table 1:** Main characteristics of the four primary schools studied

No.	Type of school	No. of teachers (ethnic minority background)	No. of classes	No. of students (male)	Ethnicity of students	Type of school latrine	Type of water supply for HW
1	Lowland branch school	5 (all Kinh)	5	89 (42)	Tày (100%)	Septic tank	Water available right outside latrine
2	Lowland main school	9 (7 Kinh and 2 Tày)	5	161 (79)	Dáy (100%)	Septic tank	Water available 20 metres away from latrines in the school yard
3	Highland branch school	9 (8 Kinh and 1 Xaphó)	5	60 (33)	Xa Phó (100%)	Septic tank	Not available (pipe water broken), used water from a stream nearby the school
4	Lowland main school	15 (13 Kinh and 2 Tày)	11	256 (116)	Tày (90%), Kinh (10%)	Septic tank	Water available, 100 metres away from latrines in school yard

### Design and data collection

The study was implemented in two phases. Phase one was a formative research phase using structured observations as well as interviews with schoolteachers, schoolchildren and their parents to understand the current teaching methods on hygiene topics in schools and to describe child hygiene behaviours (July–November 2008) (25, 28).

**Fig. 1:**
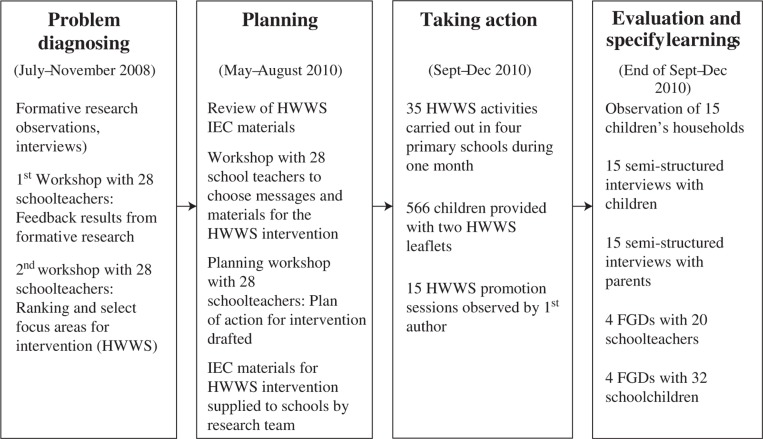
The process of an action research applied in this study.

Phase two was an education action research component (39) including a HWWS intervention at the four schools (May, September–December, 2010). This approach allowed for developing a HWWS intervention with active participation from teachers and to capture reactions to the intervention from teachers, schoolchildren and parents. The five stages of an action research as defined by O'Brien (40) were followed (Fig. 1) and included diagnosing, action planning, and taking action, evaluating and specifying learning for improvements of school-based hygiene interventions.

#### Problem diagnosis

Results from the formative research in 2008, which included thorough observations in six schools and the homes of 20 children, interviews and discussions with school staff, parents and children (25, 28) identified four hygiene behaviour problems among schoolchildren in the study communes, including limited use of latrines for defaecation, limited HWWS after defaecation and before eating, lack of access to clean drinking water and limited knowledge of the importance of maintaining personal hygiene practices. The research team organized two full-day workshops in each commune: one with all head teachers and principals and the other with all 28 schoolteachers from the two communes to discuss the constraints of improving these four hygiene behaviour problems. In the second workshop, teachers participated in ranking (41) the four hygiene behaviour constraints based on their perceptions of the feasibility to change the behaviour during a short intervention. Teachers ultimately selected HWWS as the focus of a short school-based intervention.

#### Action planning

The first author carried out a review of all available national Information, Education and Communication (IEC) materials related to HWWS for schoolchildren in Vietnam. A total of 31 HWWS-related materials were identified including various visual aids, informative materials and guidelines for HWWS. Most of the materials are from the national IEC database (42) and the Ministry of Education and Training (43).

A third workshop was facilitated in the study communes in May 2010 in which teachers at the four intervention schools were active in proposing teaching activities and how to implement them when drafting a 1-month HWWS intervention plan at their schools in corporation with the research team. Following a discussion of the reviewed teaching materials, teachers and school management in the four schools, the types of IEC materials that could be used in their interventions were selected. The materials chosen included two one-page colourful posters and two leaflets carrying the two simple messages of ‘HWWS before eating (rửa tay với xà phòng trước khi ăn)’ and ‘HWWS after defaecation (rửa tay với xà phòng sau khi đi vệ sinh)’.

#### Taking action

Schools decided to perform different types of HWWS promotional activities during school time, including in-class lectures, guidance from student's advisors during group demonstrations or talks at school meetings and by school principals during common Monday school meetings. On average, HWWS promotional activities were performed once a month in each class and for all classes during weekly school meetings over the course of 4 weeks (September–December 2010). A total of 35 of such HWWS promotional activities were carried out in the four schools over this period. All 566 children in the four schools were also provided with two copies of the leaflet on HWWS in Kinh language to take home to show their parents (Fig. 1).

#### Evaluating action and specifying learning

Several activities were carried out to capture the experiences and learning from the intervention (Fig. 1).

At school, structured observations were carried out for a total of 15 HW activities using an observation sheet including: methods used at HWWS promotion sessions, teacher's and student's reactions and duration of the activity (see Table 2). Four FGDs each with five schoolteachers and four FGDs each with eight schoolchildren were conducted after the intervention had ended in schools, to get feedback on successes and challenges faced by schoolchildren from the HWWS intervention.

**Table 2:** Observation results of HWWS intervention in schools

No.	Type of session	Educational method	School type	No. of students attended	Time for HWWS	Children response	Teacher responses
1	Monday meeting	Educational speak with poster illustration	Main, lowland	161 Dáy (grade 1–5)	Integrated (15 min)	Passive	No response
2	Monday meeting	Educational speak with poster illustration	Main, lowland	161 Dáy (grade 1–5)	Integrated (15 min)	Passive	No response
3	Monday meeting	Educational speak	Branch, lowland	89 Tày (grade 1–5)	Integrated (15 min)	Passive	Unhappy
4	Monday meeting	Short lecture, questions, rewards	Branch, lowland	20 Tày (grade 1–5)	Specific (30 min)	Active	Happy, surprised
5	School meeting	Short lecture, demonstration, practice	Branch, highland	30 XaPhó (grade 1–5)	Specific (1.5 hour)	Active, excited	Happy
6	School meeting^a^	Short lecture using posters, questions, rewards, demonstration, practice	Branch, highland	55 XaPhó (grade 1–5)	Specific (1.5 hour)	Active, excited	Happy
7	School meeting a	Power point presentation, a game, demonstration, practice	Main, lowland	251 Kinh and Tày (grade 1–5)	Specific (1.5 hour)	Active, enjoy, excited	Happy, surprised
8	Group^a^	Demonstration and practice	Main, lowland	37 Kinh and Tày (grade 3)	Specific (1 hour)	Passive	Unhappy, tired
9	Group^a^	Demonstration and practice	Main, lowland	118 Kinh and Tày (grade 4–5)	Specific (1 hour)	Active, excited	Happy
10	Lecture	Short lecture, questions, rewards	Branch, lowland	20 Tày (grade 1)	Specific (1 hour)	Active	Happy, surprised
11	Lecture	Short lecture, question, plan	Branch, Highland	11 Xa Phó (grade 4)	Specific (1 hour)	Active	Happy
12	Lecture^b^	Short lecture, poster, concrete examples	Branch, Highland	8 Xa Phó (grade 1)	Specific (1 hour)	Active	No response
13	Lecture	Short lecture, poster, concrete examples	Main, lowland	24 Kinh and Tày (grade 4)	Integrated (15 min)	Active	Happy
14	Lecture	Child group discussion	Main, lowland	12 Dáy (grade 4)	Integrated (5 min)	Passive	Unhappy
15	Lecture	Child group discussion	Branch, lowland	18 Dáy (grade 4)	Integrated (3 min)	Passive	Unhappy

a The session was conducted by a student advisor. The rest were conducted by the head teacher or the principal at weekly school meetings.

b The lecture was conducted by a male Xa Phó teacher.

During the intervention, 15 households with schoolchildren included in the intervention schools (eight 1st graders, four male and four female; seven 4th graders, three male and four female; 2 Kinh, 5 Tày, 4 Dáy and 4 Xa Phó) were selected to gain knowledge about children's and parent's responses to the HWWS intervention. Each schoolchild was observed for 5 days from the beginning of the school day at 7 AM and followed during the whole day until 8 PM at their homes. Semi-structured interviews were conducted with all 15 schoolchildren and 15 parents at their homes about the HWWS intervention and about HW at home.

### Data processing, validation and analysis

A research team including the first author and four research assistants conducted the study. Observations carried out at home and at the school in the formative phase of the study were conducted by the same research team. Observations of HWWS activities and semi-structured and open interviews with children, parents and head teachers during the intervention were all conducted in Vietnamese by the first author assisted by one research assistant seated in a private area, either at school or at home.

All semi-structured interviews and FGDs were tape-recorded and the recordings were transcribed *ad verbatim* into Vietnamese text by a research assistant. Interview and observational data were all entered and analysed using NVivo software (44). Codes were developed during the whole process of data analysis, emerging from the empirical data and inspired by concepts from literature. Main codes included: (1) hygiene teaching methods, (2) experiences with the HWWS intervention, (3) HWWS practice transfer and (4) perceived barriers to create and sustain HWWS behaviours of schoolchildren. For example, the need, but difficulties in implementing innovative and practice-based hygiene teaching methods was identified as a major theme in the literature (14, 15, 35), which was mirrored in the IEC review of this study and the empirical data from the intervention, showing teacher's wishes and challenges of implementing active methods, such as demonstrations, practice, rewards and group discussions (Table 2). Illustrative quotes presented in this article were translated from Vietnamese into English by the first author.

The study was approved by the Ministry of Health, the Ethical Committee of the National Institute of Hygiene and Epidemiology (NIHE), and by the school, commune and village authorities. In this article, all names of schools, villages and communes will be confidential; schools will be referred to by the school type (main or branches) and villages by topography (lowland or highland) to protect the anonymity of informants. Oral informed consent was given by all interviewed parents of children during the formative research and during the HWWS intervention.

## Results

### Teaching methods used in HWWS interventions

The formative research in schools showed that all teachers taught hygiene topics, including HW, according to the national curriculum using mainly theoretical and knowledge-based teaching methods, and not using any child-friendly IEC materials that could stimulate the children. Before the initiation of the action research, HWWS generally received a low priority at all schools in the study area. HWWS was taught in a 1-hour session for 1st graders under the topic of ‘Body hygiene’. The lack of focus on HWWS was further demonstrated by the fact that plans of hosting annual HWWS festivals were cancelled due to pressure on schools to focus on students’ academic performances before the annual education assessments. Observations at home further showed that families did not have the tradition of encouraging children to practice routine HWWS.

Table 2 summarizes the observations of 15 HWWS sessions at the four schools studied, including teaching methods chosen by teachers and immediate reactions by children and teachers. The experiences with teaching HWWS varied among schools, but some patterns were observed as described below.

Overall, three types of sessions were selected by the teachers including lectures; school meetings and group demonstrations (Table 2). In 10 of 15 sessions, teachers decided to adopt active teaching methods, such as competitions for rewards, demonstrations of hygiene practices, games and practice sessions into their teaching. In four of these sessions, children were visibly excited and pleased with the activities. However, 14 of the 15 sessions only had 1–3 min allocated for interactions between students and teachers. In 12 of the 15 sessions, students were observed not to have the opportunity to raise questions or discuss with the teacher.

In all sessions that involved children in performing HWWS, it was observed that the children participated actively and that it was easy to guide through the process of washing hands with little teacher guidance. There were no differences in responses of schoolchildren to these HWWS sessions based on their ethnicity and gender. However, notable differences were observed between age groups of schoolchildren, with 1st grade schoolchildren in particular being enthusiastic about the HWWS; they were observed to enjoy playing and competing for water and soap, being very fast and eager to respond to guidance on HWWS of schoolteachers, e.g. before going to class in the morning and being exited each time they lined up for HWWS. In one highland school, in particular, all schoolchildren in grade 1 performed HWWS before going to class on a daily routine basis, and approximately half of schoolchildren of grades 4 and 5 were observed to practice HWWS without being reminded, while the rest had to be reminded several times by teachers.

Another interesting finding was an observed difference in teaching methods in the smaller branch schools in the highland and the larger main schools in the lowland. Teachers in the highland schools gave more concrete and locally adapted examples on when to wash hands (e.g. after coming back home from the field, after tending the water buffalo and after playing in the school yard), which made the teaching more contextually relevant for children. Also, highland teachers gave children more opportunities for developing skills through repeated demonstrations and provided them with more frequent instructions to ensure that messages were passed on to their family.

### Evaluating HWWS interventions

Data from child interviews clearly showed that all children liked to practice HWWS irrespective of their age, ethnicity and gender. Children also gave positive responses on their feelings about the HWWS intervention at school. The main feelings expressed were comfort, excitement and being happy, while no children reported disliking HWWS. During an FGD, a group of 4th grade children explained how they felt about the HWWS intervention:

The most impressive and exciting thing in the past month was the HW-with-soap exercise, especially when our hands were clean (sạch), soft (mềm) and perfumed (thơm) …. There was nothing we did not understand …. Nothing we disliked about the exercise. (FGD with 8 grade 4 schoolchildren in highland branch)

None of the 15 parents interviewed during intervention reported any negative feeling about the intervention. They all appreciated the HWWS intervention because it corresponded well with their knowledge of good child health. According to the parents, a child with clean hands will be healthy and will not suffer from diseases:

HWWS is needed because we are afraid of dirt, disease and contamination. HWWS is good, we all know … HWWS is very essential because it helps us to prevent disease and we are poor so we are afraid of disease; if we suffer from disease, we do not have money for treatment, HWWS also helps to protect us against environmental pollution. (Interview with grade-4 male Dáy students’ parent)

Observations showed that all teachers who applied active methods in the HWWS sessions responded positively and were happy about teaching with the new methods (Table 2). Three teachers even expressed surprise that these methods created such good results and facilitated active participation by children:

The exercise went beyond my expectation as they (schoolchildren) understood quickly, and were active and gave true answers too. I am very happy and surprised! (Interview with grade 1 female Kinh teacher in lowland)

Teachers generally also expressed that they perceived it feasible to teach HWWS through school activities and that this would not overload them with additional work. Notably, no teachers mentioned that language and understanding verbal instruction was an issue in teaching HWWS for minority schoolchildren. Interestingly, teachers who applied only passive methods were observed to be dissatisfied with this type of session. When asked what they disliked about the session, they expressed that they knew other active methods that could be a better way to promote HWWS:

I don't think I have performed the session well. My expectation was not met because there was no response from the schoolchildren. (Interview with grade-4 female Kinh teacher in lowland branch)

### Transfer of information about HWWS from school to home

Home observations made during the intervention demonstrated some positive signs that schoolchildren can bring home HWWS skills and act as signs of change in their families.

Two short cases (Table 3) based on observations and interviews in a lowland home and a highland home demonstrate how children interacted with their families about HWWS.

**Table 3:** Two short cases of schoolchildren interactions with families about HWWS

A 4th grade Dáy male schoolchild from lowland family of six people:
After playing a game, the mother of Hieu aged 9 years calls him in for lunch. Hieu stands up, goes to the kitchen, fetches a small amount of washing powder from a small bag in the kitchen and c. water from a jar in the kitchen for the HW. He then washes his hands carefully following the exact steps as guided by the teacher in school. After rubbing all parts of the hands thoroughly, Hieu asks her mother to collect more water from the jar to rinse off the soap. Hieu's mother looks surprised, smiles and laughingly tells the uncle who is visiting that Hieu has learned how to wash hands on his own. Hieu asks his mother if she has washed her hands. She laughs and confirms to Hieu that she already washed her hands. Hieu proceeds indoors to ask his older brother aged 13 years if he has washed his hands. Hieu's brother now goes to the kitchen, asks his mother to also collect some water for his hand washing, he follows the steps of HWWS and finally dries his hands before going to have lunch.

A 4th grade Xa phó schoolchild from a highland family of eight people:
At 17.30 PM, the mother of Duong aged 9 years calls her in for dinner. Duong has been playing with her friends in the other households of the village all afternoon since she came home from school around lunch time. Her mother just returned from work in the fields. Duong goes to the kitchen, where water is flowing through a bamboo pipe to do HW. She finds some Vì dân powder soap in the kitchen to use. Duong says something to her mother in the local language while presenting her clean hands to the mother. During dinner, the mother explains that Duong and her younger brother of 4 years have been practicing HW much more often than usually during this week (when HWWS intervention was introduced at school). She is always away from home during daytime to do work in the fields in the hills and cannot remind the children to wash hands. She thinks it is really good that Duong and her brother can wash hands on their own now.

From the two cases and other families, it was found that children's new knowledge on HWWS was typically shared with families through social interactions and conversations before or during meals (7 of 15 children) when families gathered. In the 15 families visited, six schoolchildren were observed to wash their hands before having lunch or dinner automatically, while two children washed after cues from siblings, and five needed cues by parents or grandparents and two of them did not wash at all. Observations also indicated some differences between young and older schoolchildren; while 1st graders were able to practice HWWS on their own at home, they did not convey any of the verbal information from teachers and lectures to their families. Older students such as the two described in Table 3, were observed to practice HHWS and also convey verbally HWWS knowledge to their families, request siblings to do HWWS and parents to provide soap for hand washing. A mother of a 4th grade female Tày student explained how the intervention had brought her new knowledge on health and HW through her child:

The HW information is of interest to us because we know from television that if children are clean, they will sleep well and grow faster. The schoolchildren also mentioned that daily HW makes you healthy. In the past, we did not have the opportunity to go to school. When we were the same age as our children we did not have the same level of knowledge. But now the children learn a lot from school also things which is new to us.

### Perceptions on practicing and sustaining HWWS practices

During interviews at homes, all parents expressed a willingness to reinforce HWWS. Observations suggested that there were no infrastructural barriers to HWWS; all 15 households had adequate soap and water available for performing HWWS. The type of water at household level included gravity and water tank. In addition, all interviewed parents stressed that they perceived various types of soaps as being suitable for HW in their homes (e.g. dishwashing liquid or washing powder) and that they had no problems in always having soap available for HW:

Sometimes, my house doesn't have bar of soap (xà phòng) so we use powder soap (xà phòng giặt) or dishwashing liquid (nướuc rủa bát). (Interview with the mother of a grade 1 female schoolchild in highland)

A total of 14 of 15 schoolchildren interviewed stated that they did not find it difficult to practice HWWS at home and none of them mentioned lack of water or soap or lack of advice as barriers for practicing HWWS.

This was in contrast to all interviewed schoolteachers, who felt that major investments were necessary for water supply, HW facilities and soap (in homes as well as schools), if teaching HWWS should be effective and sustainable. However, during the intervention, it was observed that HWWS was easily taught in all four schools independent of their level of water supply and availability of HW stations. Teachers also perceived the poverty of communities as an important barrier for creating new child hygiene habits, particularly in the highland. They also thought that parents did not take enough interest in encouraging HWWS at home:

The economic conditions of many households are difficult (Xa Phó group), so they still do not have soap and water for washing hands. There is also a lack of interest from the family. Teachers remind students only when they are at school but they may forget when they go back home because no one reminds them there. (FGD with 5 Kinh teachers in a highland school)

This was corroborated to some extent by observations in homes; schoolchildren in the highland clearly received less parental guidance on many aspects of care and health including personal hygiene and HWWS compared with children from the lowland areas. One explanation was that highland parents were often away from early mornings to evenings doing fieldwork, which left children in the care of other children (see case 2 described in Table 3).

Finally, all schoolteachers participating in FGDs doubted the feasibility of continuing and sustaining HWWS behaviour after the end of the intervention. They mentioned longer intervention periods with more frequent reminders are necessary to change children's habits:

Changes were seen since students washed their hands with water before eating, and after using the toilet. However, they do not always wash regularly, so we need more time because the children easily forget (hay quên). (FGD with 5 Kinh female teachers in lowland branch school)

## Discussion

This action research investigated and identified opportunities and constraints in implementing new teaching methods for changing HWWS practices and hygiene behaviour of schoolchildren in a multi-ethnic population. The low priority and low intensity of teaching hygiene behaviours at school along with lack of parental guidance in particular highland communities seem to present key barriers likely to hamper HWWS behaviour change and its sustainability. These barriers will be further discussed below.

### Teaching methods to change hygiene behaviour

The effect of school hygiene promotion through participatory and practical methods has been studied in developed (45) as well as in developing countries (15, 35–37, 46). These studies have indicated that practical exercises and approaches can change schoolchildren's hygiene behaviour (35, 46) and that children and teachers are enthusiastic about action-oriented lessons due to clear goals and observable results (36). A study among primary schoolchildren in Kenya specifically highlighted that action-oriented health education can develop increased ownership of the new knowledge and thereby transform children's behaviour through their participation (47).

In Vietnam, there is a growing criticism of the traditional knowledge-based teaching approaches used in schools. Such criticism has led to an increased focus on child-centred learning in educational reforms done by the Ministry of Education and Training in the past two decades (48). Schools and other implementers are required by the Ministry both to ‘restructure’ and ‘reculture’, including a redesigning of curricula and renewal of teaching and learning styles, e.g. allowing more time for interaction between teachers and schoolchildren (49). Despite this new high priority, the present and other school-based studies of multi-ethnic populations have highlighted that teachers still apply teaching styles focusing upon knowledge transfer and with close teacher control (50)—even when teaching practical issues such as HWWS.

The reasons for this dominating didactic teaching approach may partly be explained by Vietnam's education history originating from Confucian philosophy (51, 52). Several studies of higher education institutions in Vietnam have documented that, like in other Asian countries, learners according to Confucian philosophy have strong beliefs in teacher's authority and have little experience in self-learning or studying independently (53, 54). This was also supported by an anthropological study of child upbringing norms in the northern Vietnam, where Rydstrøm found that teachers perceived children as being like ‘white pieces of paper’ who need to passively absorb information from adults. However, Rydstrøm also observed that schoolchildren were indeed active in their own learning process through daily social interaction with siblings, peers and adults (55). In agreement with the study of Rydstrøm, we argue that schoolchildren in multi-ethnic populations can learn good hygiene behaviour, not through passive teachings in schools, but through daily practical interactions across several social settings with peers, family members and teachers. It is therefore highly recommended to develop and implement a skill-based and participatory teaching curricula and teaching culture in schools of Vietnam, if schools and schoolteachers are expected to support changes in child hygiene and HWWS behaviour.

Few, simple and clear messages have been shown as most effective for hygiene education (56). A study of higher education found that short supply of material sources and lack of reference books would hamper the application of child-centred teaching methods in schools of Vietnam (57). The review of suitable HWWS materials leading up to the present HWWS intervention revealed a lack of suitable materials to be used to facilitate and discuss HWWS. Future IEC hygiene materials for schoolchildren should be developed. These should be colourful and convey few and clear messages in order for all children and families to understand, regardless of literacy and language. The communication materials could be posters, flip charts, flash cards, wall murals, puzzles and teaching guides, that were found to successfully change hygiene behaviour in schools and communities in rural Bangladesh (58).

### Investing in hygiene education

A study of higher education in Vietnam identified some barriers for implementing student-centred teaching methods, including a heavy teaching curriculum and as found in this study, a lack of time for student-driven activities (57). A child-to-child, quasi-experimental study in rural Kenya using a participatory and action-oriented approach to hygiene education found that extra time, cost and manpower were needed if active teaching on malaria, diarrhoea and hygiene were to be successful (15). This is in contrast to this study where we demonstrated that despite some perceived barriers among teachers, educational activities to improve HWWS behaviour could be performed without major investments in infrastructure, financial support and time-consuming preparations. Other studies have furthermore stressed that HW interventions can be initiated at any step on a ‘HW ladder’ moving from simple HW with water, towards HW with soap, and still be effective (9). A number of low-cost technical facilities to support HWWS have also been developed, including HW stations, which can be considered for institutional settings with poor school water supply (59).

We clearly acknowledge that facilities such as access to water and soap are important barriers to good HWWS practices for schoolchildren (25, 28). However, even with water and soap available it is clear that software inputs such as behaviour change interventions are still needed to establish sound practices. Thus, we argue that HWWS interventions may not demand large infrastructural investments and more human resources, but will definitely need ambitious investments into creating ‘HWWS cultures’ and behaviour change of children at schools. School managements and educational authorities must therefore prioritise and invest in training of skill-based teaching methods and motivate teachers to apply them.

### Sustainability and transfer of hygiene behaviour

Behavioural studies on hygiene in small children have shown that hygiene behaviour such as HW is highly habitual, and formed on the basis of learned automated behaviour formed from an early age. It needs repeated practice to become established as a routine. Therefore, educational efforts also have to start early in life (1, 60).

Several organisations, including UNICEF, have mentioned children as important agents of change for health when they bring new knowledge and skills back home (13–20). However, UNICEF also stresses that schools and teachers need to play vital roles as advocators for change by encouraging children to share with their families and communities – influencing children only in schools is simply not enough to create new hygiene habits (18). Hence, a school-based hygiene education in rural Kenya found that 14 months after intervention, there was a remarkable renovation of HW facilities at home, after children had actively encouraged families to wash their hands (13).

This study provided important insights into some key constraints in sustaining hygiene behaviour in schoolchildren in low resource settings which needs to be addressed in future hygiene programmes. First, a lack of parental guidance for highland children in particular due to agricultural workloads, seemed to constrain the abilities of parents to establish HWWS at home as a routine behaviour early in life. Furthermore, schools in the study area had not included HWWS in their curricula and even during this short intervention. HWWS messages were given once a week maximum, and in some cases through passive teaching methods. Finally, schools did not actively engage families or communities but relied on children of all ages to convey the hygiene messages to their homes.

However, as documented in this study the ability of small children (1st graders) to transfer their hygiene knowledge is not likely. The main reason for a failure in the transfer of HWWS messages from schools to homes by small children seems to be due to the lack of communication between schools, communities and families. Issues of power relations between majority and minority groups, poverty, cultural differences and language barriers are also likely to have influenced communications but have not been investigated in great detail as part of this study. Hence, we conclude that low-intensity hygiene interventions relying on individual and linear transfer of knowledge and skills from student to family is not likely to be a successful strategy to change and sustain new hygiene behaviour in this and similar contexts. Wider social interventions are indeed needed to ensure that HWWS and related hygiene practices become a social norm valued by communities, including close corporation between schools, communities and parents. To achieve this, high political priority of hygiene as an important community intervention area is also necessary.

### Limitations of the study

This study had some important limitations. First, it is proposed that a minimum of three cycles of repeated interventions are required for a full action research study to adjust the intervention appropriately to participants and the social context, create sustainable behaviour changes and establish ownership of the intervention by participants (61). This intervention study was conducted only once and over a short period of a single month, due to time constraints. This has most likely decreased the ability of the teachers to establish ownership and optimize the teaching methods and continue the hygiene promotional activities after the end of researcher-facilitated intervention. In addition, it was short-termed and only focused on teachers without targeting parents and other household members. Despite these limitations, the study did draw out important learning on potentially effective active teaching methods for HWWS for children of different ages and settings, which can be further developed in future school hygiene programmes.

Second, the observation effect resulting from the presence and interaction with the research team could have increased the motivation of schoolchildren and teachers to change HWWS behaviour during the intervention (62), which could portray an overly optimistic response to a HWWS intervention. This might have been exacerbated by the fact that the study area had been quite heavily exposed to hygiene and HWWS messages following the Avian Flu outbreaks in Vietnam in 2008.

Third, the intervention was carried out only in four schools and included only one EMG in a highland setting (Xa Phó). However, living conditions in other large EMGs in highland settings such as the Red Dao and H'Mongs can be expected to be similar or of a lower socio-economic status since they often live in remote mountainous villages without access to public services (63).

## Conclusions

The engagement of schoolteachers in teaching HWWS practices to primary schoolchildren shows promises to improve child hygiene behaviour without the need for major investment in water and hygiene infrastructures. However, hygiene behaviour of schoolchildren cannot be expected to change unless schools develop and implement practical teaching methods and that health promotion programmes have a stronger focus on creating HWWS routines in schools as well as in homes. Further studies to assess long-term behaviour change following HWWS intervention and development of innovative teaching methods are warranted.
